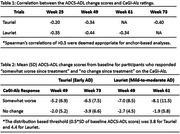# Deriving meaningful within‐patient change thresholds for the ADCS‐ADL in trials of early and mild‐to‐moderate Alzheimer’s disease ‐ a caregiver‐rated, anchor‐based analysis of the Tauriel and Lauriet trials

**DOI:** 10.1002/alz.084155

**Published:** 2025-01-09

**Authors:** Marina Ritchie, Seema Datta, Claire J Lansdall, Cecilia Monteiro, Balazs Toth, Edmond Teng

**Affiliations:** ^1^ Genentech, Inc., South San Francisco, CA USA; ^2^ F. Hoffmann‐La Roche Ltd, Basel Switzerland

## Abstract

**Background:**

Clinical outcome assessments (COAs) that measure functional capacities are key tools to evaluate efficacy in Alzheimer’s disease (AD) clinical trials. The Alzheimer’s Disease Cooperative Study Activities of Daily Living (ADCS‐ADL) scale is frequently used to assess changes in both basic and instrumental activities of daily living, but there is no clear consensus on what magnitude of change on this scale may be considered clinically meaningful. To address this question, we conducted anchor‐based analyses (as recommended by the FDA) to explore meaningful within‐patient/participant change thresholds on the ADCS‐ADL.

**Methods:**

Our anchor‐based analyses (and supportive distribution‐based analyses) used data from two phase 2 semorinemab trials, Tauriel (NCT03289143; early AD) and Lauriet (NCT03828747; mild‐to‐moderate AD). The Caregiver Global Impression of Change‐Alzheimer’s Disease (CaGI‐Alz), a caregiver‐reported measure on subjective change in participant function, served as the anchoring scale. To determine if anchor‐based analyses were appropriate, we first examined correlations between the ADCS‐ADL change scores and CaGI‐Alz ratings at Weeks 25 and 49 for both trials, along with Week 61 for Lauriet, and Week 73 for Tauriel. To determine meaningful change thresholds, we analyzed the mean changes on the ADCS‐ADL for participants rated as performing “somewhat worse” on CaGI‐Alz. Those rated as demonstrating“no‐change” were used for comparison.

**Results:**

Correlations between the ADCS‐ADL and the CaGI‐Alz scales were consistently stronger at Week 49 and later time points (Table 1). For those rated as “somewhat worse,” mean decline on the ADCS‐ADL ranged from approximately 5 to 8 points across time points, and uniformly exceeded distribution‐based thresholds. Larger declines on the ADCS‐ADL were associated with “somewhat worse” ratings in patients in Lauriet (mild‐to‐moderate AD) compared to Tauriel (early AD) and at later time points in both trials (Table 2). Patients rated as “no change” by the caregiver experienced some decline on the ADCS‐ADL, although generally less than the declines seen in those rated “somewhat worse.”

**Conclusion:**

The anchor‐ and distribution‐based approaches suggest that clinically meaningful change on the ADCS‐ADL may be greater than the 2 points that has previously been postulated (Dysken et al., 2014). Higher change thresholds may be required to reflect meaningful changes perceptible to caregivers.